# Metabolic Engineering of *Candida glabrata* for Diacetyl Production

**DOI:** 10.1371/journal.pone.0089854

**Published:** 2014-03-10

**Authors:** Xiang Gao, Nan Xu, Shubo Li, Liming Liu

**Affiliations:** 1 State Key Laboratory of Food Science and Technology, Jiangnan University, Wuxi, Jiangsu, China; 2 Key Laboratory of Industrial Biotechnology, Ministry of Education, School of Biotechnology, Jiangnan University, Wuxi, Jiangsu, China; 3 Laboratory of Food Microbial-Manufacturing Engineering, Jiangnan University, Wuxi, Jiangsu, China; University of Nottingham, United Kingdom

## Abstract

In this study, *Candida glabrata*, an efficient pyruvate-producing strain, was metabolically engineered for the production of the food ingredient diacetyl. A diacetyl biosynthetic pathway was reconstructed based on genetic modifications and medium optimization. The former included (i) channeling carbon flux into the diacetyl biosynthetic pathway by amplification of acetolactate synthase, (ii) elimination of the branched pathway of α-acetolactate by deleting the *ILV5* gene, and (iii) restriction of diacetyl degradation by deleting the *BDH* gene. The resultant strain showed an almost 1∶1 co-production of α-acetolactate and diacetyl (0.95 g L^−1^). Furthermore, addition of Fe^3+^ to the medium enhanced the conversion of α-acetolactate to diacetyl and resulted in a two-fold increase in diacetyl production (2.1 g L^−1^). In addition, increased carbon flux was further channeled into diacetyl biosynthetic pathway and a titer of 4.7 g L^−1^ of diacetyl was achieved by altering the vitamin level in the flask culture. Thus, this study illustrates that *C. glabrata* could be tailored as an attractive platform for enhanced biosynthesis of beneficial products from pyruvate by metabolic engineering strategies.

## Introduction

Diacetyl is known to be the major component in butter [1], and is widely used in artificial butter flavoring, margarines, or similar oil-based products to impart butter flavor to the final product. When compared with diacetyl production through chemical synthesis, microbial production of diacetyl is more safe and environmental-friendly. Various prokaryotic organisms such as lactic acid bacteria and *Enterobacter aerogenes* have been studied for their potential role in diacetyl production (Table 1). In these bacteria, acetolactate synthase (ALS) catalyzes the condensation reaction of pyruvate to α-acetolactate, which is subsequently converted to diacetyl through non-enzymatic decarboxylation (NOD) in the presence of oxygen. The intermediate α-acetolactate can be channeled to acetoin through acetolactate decarboxylase (ALDB). In addition, diacetyl can be converted to acetoin and 2,3-butanediol in a two-step reduction process by diacetyl reductase (DR) and butanediol dehydrogenase (BDH) [2].

**Table 1:** Comparison of diacetyl production using different microorganisms in terms of culture medium, engineering strategies, and performance.

Organisms	Year	Culture medium	Engineering strategies	Diacetyl (g L^−1^)	Yield (mol mol^−1^)	Reference
*Lactobacillus casi*	2009	MRS	Deletion of LDH and the E2 subunit of PDHc, overexpression of ALS	1.4	0.15	[3]
*Lactococcus lactis*	2000	Potassium phosphate buffer with 0.5% glucose	Overexpression of NOX, deletion of ALDB	0.38	0.16	[6]
*Lactococcus lactis*	2012	Reconstituted skim milk with 1% glucose	Overexpression of NOX, deletion of ALDB	0.36	0.07	[7]
*Lactococcus lactis subsp. latis* biovar *diacetylactis*	2000	MRS	Attenuation of LDH and ALDB	0.52	0.05	[4]
*Enterobacter aerogenes*	2009	Chemically defined medium	A UV mutant with decreased activities of ALDC, LDH and DR	1.35	0.04	[5]
*Streptococcus thermophilus*	2007	Reconstituted skim milk	Inactivation of ALDB	0.02	-	[8]
*Candida glabrata* DA-3	2013	Chemically defined medium	Overexpression of ALS, deletion of AHAIR and DR, medium optimization	4.70	0.10	This study

Several metabolic engineering strategies have been designed to improve diacetyl production (Table 1). To increase the carbon flux toward the α-acetolactate pathway for diacetyl production, ALS was overexpressed and the pyruvate alternative pathways were blocked [3]. In addition, a combined inactivation of ALDB was found to be necessary to reroute the metabolic flux from acetoin to diacetyl synthesis. Monnet et al. showed that *Lactococcus lactis* mutants with low ALDB and lactate dehydrogenase (LDH) activities were able to overproduce diacetyl and α-acetolactate [4]. In another study, a mutant strain of *E. aerogenes* with decreased ALDB, LDH, and DR activities was developed, and a maximum diacetyl titer of 1.35 g L^−1^ was achieved [5]. Furthermore, NADH oxidase (NOX) overexpression *in L. lactis* was reported to be an efficient strategy to reroute the metabolic flux away from lactate production toward oxidized products [6], [7]. Moreover, a combination of NOX overexpression with ALDB inactivation in *L. lactis* was observed to reroute 80% of pyruvate produced from glucose breakdown to diacetyl synthesis via α-acetolactate [6]. However, most of these studies had been based on lactic acid bacteria [3], [6]–[8], and were aimed at conferring intense butter aroma to products such as cheese and yogurt. Although the diacetyl yields of some engineered strains have been impressive, the final diacetyl titers have been low for efficient purification and large-scale diacetyl production (Table 1).

The multivitamin auxotrophic yeast *Candida glabrata* CCTCC M202019 is a well-established microorganism used for the industrial production of pyruvate [9]. The biosynthetic pathway of diacetyl in *C. glabrata* is similar to that in other bacteria. However, it was identified that *C. glabrata* does not produce the enzyme ALDB [10], which competes with diacetyl for α-acetolactate. Moreover, it was found that the concentrations of thiamine (cofactor for pyruvate dehydrogenase complex (PDHc) and pyruvate decarboxylase (PDC)), nicotinic acid (NA; cofactor for glycolysis and PDHc), pyridoxine, and biotin (cofactor for pyruvate carboxylase) in the medium could affect the corresponding enzymes [9]. It has been reported that under optimal concentration of vitamins, *C. glabrata* could produce byproducts at low level and block most of the carbon flux at the node of pyruvate (*C. glabrata* can accumulate pyruvate extracellularly with a yield of 0.635 g (g glucose)^−1^) [11], a key precursor of diacetyl. These advantages make *C. glabrata* a promising alternative host for diacetyl production.

**Figure 1:**
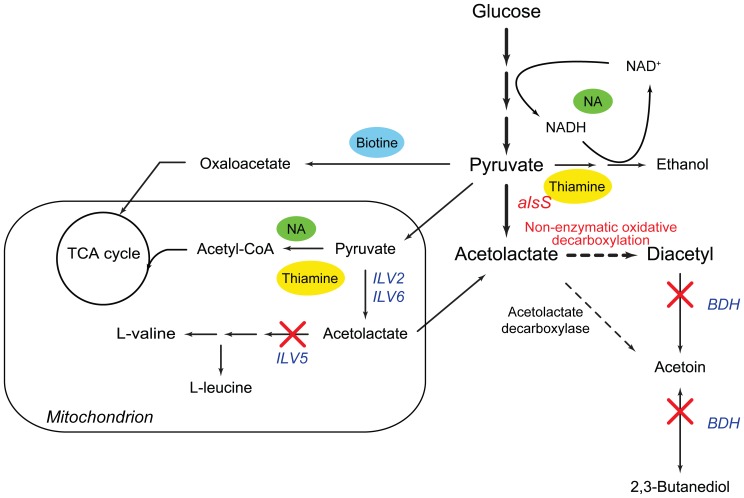
Illustration of diacetyl metabolic pathway and pyruvate metabolism in engineered *C. glabrata*. The red marks indicate metabolic modifications performed in this study, and the cytosolic ALS encoded by *alsS* was from *B. subtilis*. Additional descriptions of the reactions with thiamine and NA as cofactors are provided in Table S1.

In this study, *C. glabrata* CCTCC M202019 was tailored for diacetyl production (Figure 1). With the aim of redirecting the carbon flux to diacetyl from the pyruvate node, the accumulation of α-acetolactate was first enhanced through overexpression of the ALS encoded by the *alsS* gene from *Bacillus subtilis*. Then, the branched pathway of α-acetolactate and degradation of diacetyl were eliminated by deleting the genes *ILV5* and *BDH*, respectively. The addition of metal ions to the medium enhanced the NOD of α-acetolactate to diacetyl, and re-optimization of the vitamin levels was an essential process to achieve increased diacetyl production.

## Results

### Overexpression of ALS Leads to Diacetyl Production

**Figure 2:**
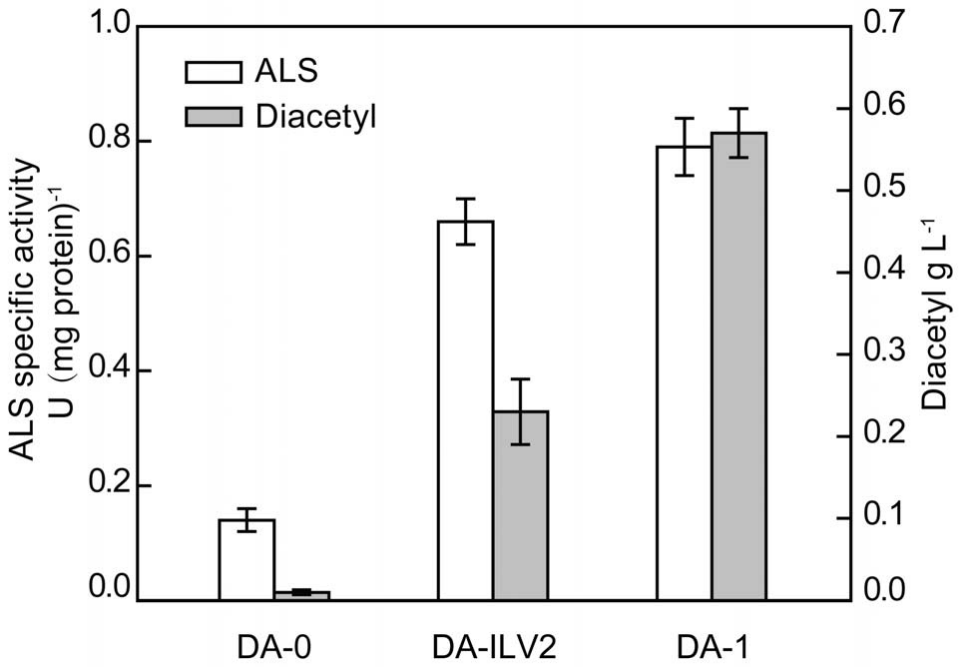
Evaluation of ALS activity and diacetyl production using different ALS enzymes. The ALS activities were determined in cells at mid-log growth phase of batch-flask fermentation. The mean values of three independent experiments are shown. The error bars indicate the respective standard deviations.

To select the right ALS as the overexpression target for diacetyl production, two different ALS enzymes were introduced into *C. glabrata*, respectively. First, overexpression of the native ALS encoded by the *ILV2* gene resulted in a 3.7-fold increase in ALS activity and increased the diacetyl production to 0.23 g L^−1^ from trace amounts, when compared with the control strain DA-0 harboring empty plasmid (Figure 2). Alternatively, the strain overexpressing the *alsS* gene encoding ALS obtained from *B. subtilis* showed a slight increase in the ALS activity and could produce 0.57 g L^−1^ of diacetyl, when compared with that overexpressing the *ILV2* gene (Figure 2). Therefore, the *alsS* gene was selected for overexpression and the corresponding engineered strain was designated as DA-1.

**Figure 3:**
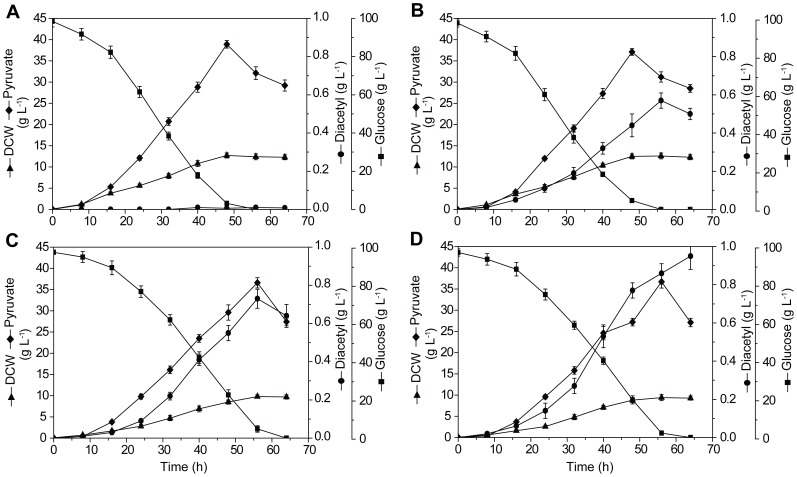
Fermentation profile for cell growth and product accumulation during shake-flask culture of the control strain DA-0 (A) and the engineered strains DA-1 (B), DA-2 (C), and DA-3 (D).

The fermentation parameters of the engineered strain DA-1 and control strain DA-0 were compared. First, the growth rate of DA-1 and its biomass concentration were noted to be slightly lower than those of DA-0 (Figure 3A and 3B). In strain DA-0, there was almost no carbon flux in the diacetyl metabolic pathway (Table 2). In contrast, with increased ALS activity in strain DA-1, the ability to accumulate α-acetolactate was significantly upregulated, leading to diacetyl production. The final α-acetolactate and diacetyl productions were 0.61 and 0.57 g L^−1^, respectively. In addition, accumulation of α-acetolactate also resulted in the increased productions of L-valine, acetoin, and 2,3-butanediol, suggesting increased carbon fluxes in the branched pathway of α-acetolactate and degradation pathway of diacetyl (Table 2). In response to the diversion of carbon flux into α-acetolactate pathway, strain DA-1 showed a 4.7% decrease in pyruvate production, when compared with strain DA-0, and the ethanol accumulation level remained constant (Table 2).

**Table 2:** Comparison of fermentation results obtained with metabolically engineered *C. glabrata* strains and the control strain.

Strains	Pyruvate	Acetolactate	Diacetyl	Acetoin	2,3-Butanediol	Valine	Leucine	Ethanol
DA-0	38.9±0.7^a^	0.01±0.00	0.01±0.00	0.03±0.00	0.03±0.01	0.02±0.00	0.01±0.00	1.76±0.14
DA-1	37.1±1.0	0.61±0.06	0.57±0.07	0.21±0.03	0.23±0.04	0.03±0.00	0.00±0.00	1.69±0.09
DA-2	36.6±1.2	0.83±0.07	0.73±0.05	0.23±0.02	0.28±0.02	–	–	1.86±0.12
DA-3	36.7±1.5	0.86±0.05	0.95±0.07	0.11±0.00	0.08±0.00	–	–	1.80±0.07

a Concentration of fermentation products (g L^−1^).

### Host Gene Deletion to Increase Diacetyl Production

**Figure 4:**
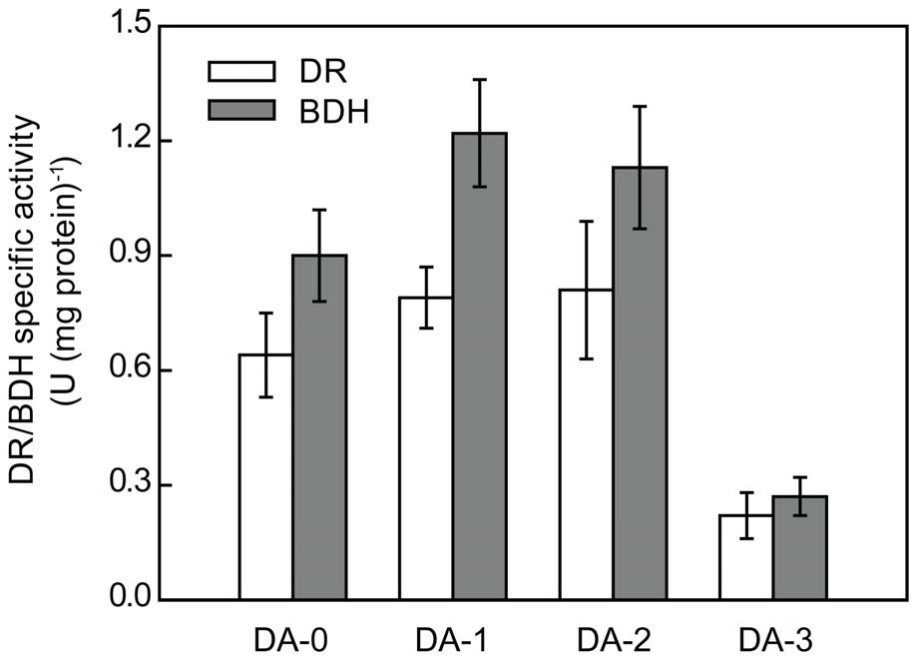
Comparison of DR and BDH activities in the engineered strains and control strain. The activities of DR and BDH were determined in cells at mid-log growth phase of batch-flask fermentation. The mean values of three independent experiments are shown. The error bars indicate the respective standard deviations.

The *ILV5* gene, encoding acetohydroxy acid reductoisomerase, was deleted in the strain DA-1 to form DA-2. As shown in Table 2, strain DA-2 exhibited 28% higher diacetyl production and 37% higher α-acetolactate accumulation than DA-1. The deletion of *ILV5* also led to a slight increase in the accumulation of acetoin and 2,3-butanediol. To produce strain DA-3, the *BDH* gene was deleted in strain DA-2. Although deletion of the *BDH* gene could not completely disrupt the activities of DR and BDH (Figure 4), it did increase diacetyl production by lowering acetoin formation from 0.23 to 0.11 g L^−1^ and 2,3-butanediol formation from 0.28 to 0.08 g L^−1^ (Table 2). The strain DA-3 showed an almost 1∶1 co-production of α-acetolactate and diacetyl (0.95 g L^−1^). Table 2 also shows that the changes in pyruvate and ethanol concentrations of the cultures of strains DA-2 and DA-3 were not substantial (<10%), when compared with those of their respective controls. These results indicated that the deletion of *ILV5* and *BDH* genes effectively redirected the flux at the α-acetolactate and diacetyl node, but had no apparent effect on the flux distribution around the pyruvate node.

It should be noted that the deletion of *ILV5* introduced auxotrophy for branched-chain amino acids and D-pantothenate. With the addition of sufficient nutrients, the growth rate of strain DA-2, with a value of 0.12 h^−1^, was lower than that of DA-1 (0.15 h^−1^), and the biomass concentration of DA-2 was 81.2% of that of DA-1 (Figure 3B and 3C). In addition, strain DA-2 also showed lower glucose consumption rate, when compared with strain DA-1 (Figure 3B and 3C). On the other hand, the additional deletion of *BDH* in DA-2 had no effect on the growth rate and biomass concentration, and the glucose consumption rates of strains DA-2 and DA-3 were identical (Figure 3C and 3D).

### Fe^3+^ Enhanced NOD of α-Acetolactate to Diacetyl

**Figure 5:**
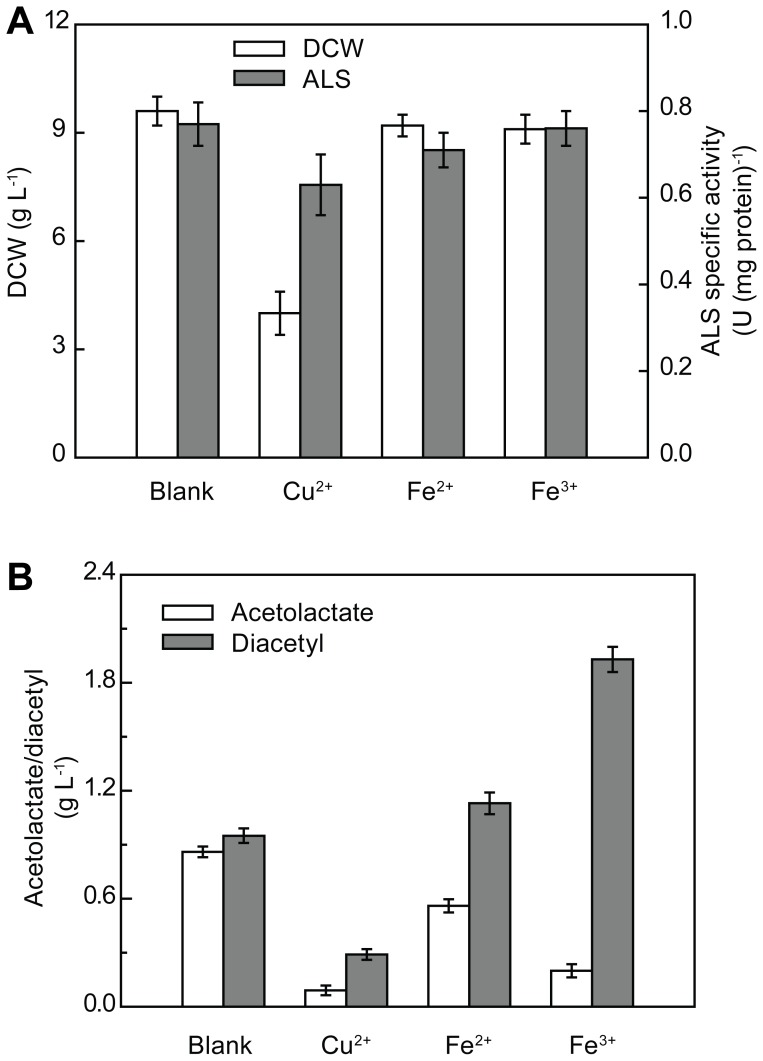
Characteristics of the engineered strain DA-3 in medium containing Cu^2+^, Fe^2+^, and Fe^3+^. (A) Effects of metal ions on cell growth and ALS activity. (B) The ability of metal ions to transform α-acetolactate to diacetyl in the fermentation process. The present data were obtained after optimizing the titers of the metal ions added with respect to diacetyl production and cell growth.

In order to achieve optimal production of diacetyl alone, rather than co-production of α-acetolactate and diacetyl, NOD of α-acetolactate to diacetyl should be enhanced. Mohr et al. [12] reported that α-acetolactate can be converted to diacetyl by heating in the presence of metal ions (Cu^2+^, Fe^2+^, Fe^3+^, and Mo^6+^) or hemin. In the present study, the characteristics of the engineered strain DA-3 in medium containing Cu^2+^, Fe^2+^, and Fe^3+^ were studied. Fe^3+^ was selected as an accelerator for the decarboxylation of α-acetolactate to diacetyl, because the addition of Fe^3+^ exhibited lower inhibition for strain growth as well as ALS activity, but preferable oxidant activity, when compared with Cu^2+^ and Fe^2+^ (Figure 5). As shown in Table 3, addition of 20 mM Fe^3+^ led to a two-fold increase in diacetyl production up to a level of 2.1 g L^−1^ and decreased the accumulation of α-acetolactate by 79.1%. Furthermore, Fe^3+^ addition also resulted in 75 and 62.5% increase in the accumulation of acetoin and 2,3-butanediol, respectively. These results suggested that the NOD of α-acetolactate to diacetyl could be enhanced by the addition of Fe^3+^.

**Table 3:** Results of fermentation carried out using strain DA-3 with different concentrations of metal ions and vitamins.

Metal ions/vitamins levels	Control^a^	A	B	C
Consumed glucose (g L^−1^)	96.9±1.9	96.4±2.5	96.8±3.6	97.3±2.7
Specific growth rate (1 h^−1^)	0.12±0.01	0.12±0.02	0.19±0.03	0.13±0.01
Biomass (g L^−1^)	9.3±0.05	9.3±0.4	15.3±0.7	11.2±0.4
Diacetyl (g L^−1^)	0.95±0.07	2.1±0.2	3.5±0.2	4.7±0.4
α-Acetolactate (g L^−1^)	0.86±0.05	0.18±0.02	0.25±0.01	0.24±0.03
Acetoin (g L^−1^)	0.11±0.00	0.18±0.01	0.51±0.03	0.32±0.02
Butanediol (g L^−1^)	0.08±0.00	0.15±0.02	0.36±0.02	0.61±0.05
Yield (g (g glucose)^−1^)^b^				
Diacetyl	0.01	0.02	0.04	0.05
Ethanol	0.02	0.02	0.12	0.13
Biomass	0.10	0.10	0.16	0.12
Pyruvate	0.38	0.36	0.16	0.10

a Control: 0 mM FeCl_3_, 0.02 mg L^−1^ VB_1_, 8 mg L^−1^ NA.

A: 20 mM FeCl_3_, 0.02 mg L^−1^ VB_1_, 8 mg L^−1^ NA.

B: 20 mM FeCl_3_, 0.08 mg L^−1^ VB_1_, 8 mg L^−1^ NA.

C: 20 mM FeCl_3_, 0.08 mg L^−1^ VB_1_, 2 mg L^−1^ NA.

VB_1_ and NA represent thiamine and nicotinic acid, respectively.

b The standard deviation for each yield was below 10%.

### Effect of Thiamine and NA levels on Carbon Flux Redistribution

The concentrations of thiamine, NA, pyridoxine, and biotin in the medium were minimized to limit the activities of enzymes responsible for further conversion of pyruvate and achieve increased accumulation of pyruvate. Therefore, we examined whether low concentration of thiamine, the cofactor for ALS (Table S1) [13], limited the production of diacetyl. As shown in Table 3, with the supplementation of 0.08 mg L^−1^ of thiamine to the medium, a 3.5-g L^−1^ diacetyl production was achieved, which was 81.3% higher than that of the control (0.02 mg L^−1^ of thiamine). The concentrations of acetoin and 2,3-butanediol were also found to increase from 0.18 to 0.51 g L^−1^ and from 0.14 to 0.36 g L^−1^, respectively. Furthermore, strain DA-3 showed obvious increase in the growth rate and biomass as well as in the accumulation of ethanol. The biomass and ethanol yields increased from 0.09 to 0.15 g (g glucose)^−1^ and from 0.02 to 0.12 g (g glucose)^−1^, respectively. As a result, the accumulated pyruvate titer decreased to 15.9 g L^−1^ with a yield of 0.17 g (g glucose)^−1^ (Table 3). These results suggested that increased carbon flux was redirected into the TCA cycle and ethanol pathway, in addition to the diacetyl biosynthetic pathway, in the presence of higher concentration of thiamine.

Subsequently, the effect of NA, another cofactor involved in the TCA cycle and ethanol pathway, but not in the diacetyl biosynthesis (Figure 1 and Table S1), on diacetyl production was evaluated. As shown in Table 3, with the decrease in the NA level from 8 to 2 mg L^−1^, the strain DA-3 first showed a decrease in both growth rate and biomass. However, contrary to the expectation, the yield of ethanol was increased by 13.5%. This increase could be elucidated by the improved NADH/NAD^+^ ratio from 0.53 to 0.79 (Table 4), suggesting a more reductive intracellular environment. The final diacetyl titer was 4.7 g L^−1^, which was 34.3% higher than that obtained in the control. Furthermore, the yield of pyruvate was further decreased to 0.12 g (g glucose)^−1^ (Table 3).

**Table 4:** Comparison of intracellular NADH/NAD^+^ concentrations of strain DA-3 at different vitamin levels.

Vitamin levels	A^a^	B	C
Intracellular NAD^+^ (µmol (g DCW)^−1^)	7.3±0.7	7.8±0.6	4.2±0.3
Intracellular NADH/NAD^+^ ratio	0.55	0.53	0.79

a A: 0.02 mg L^−1^ VB1, 8 mg L^−1^ NA.

B: 0.08 mg L^−1^ VB1, 8 mg L^−1^ NA.

C: 0.08 mg L^−1^ VB1, 2 mg L^−1^ NA. VB_1_ and NA represent thiamine and nicotinic acid, respectively.

## Discussion

Due to the excellent performance of *C. glabrata* as an industrial pyruvate-producing strain, the research on redirecting carbon flux toward the desired pathways and developing a platform strain for the production of other useful bioproducts is quite attractive. In the present study, we demonstrated the successful metabolic engineering of *C. glabrata* for enhanced production of a target product, using diacetyl as an example. Three metabolic modifications contributed to increased production of diacetyl: (i) directed genetic modification of diacetyl biosynthetic pathway; (ii) enhanced conversion of α-acetolactate to diacetyl in the presence of Fe^3+^; and (iii) redistribution of the carbon flux around the pyruvate node by manipulating the cofactor (thiamine and NA) levels.

In most of the reported studies, the pyruvate alternative pathway was inactivated to improve diacetyl production (Table 1). In *C. glabrata*, the carbon flux was blocked at the pyruvate node, which allowed channeling of the carbon flux into diacetyl biosynthetic pathway by direct overexpression of ALS. Two different types of ALS have been reported to exist in microorganisms. One is involved in the biosynthesis of branched-chain amino acids, and its overexpression had been used for enhanced production of diacetyl in *Lactobacillus casei* or for the production of L-valine in *Escherichia coli* [3], [14]. The other type of ALS is a catabolic enzyme required for 2,3-butanediol fermentation, and had been employed for the robust production of acetoin or 2,3-butanediol in prokaryotic strains such as *B. subtilis*, *Bacillus amyloliquefaciens*, and *Klebsiella oxytoca* [15]–[17]. In a recent study, the ALS of *B. subtilis*, encoded by the *alsS* gene, was overexpressed for improved 2,3-butanediol production in *Saccharomyces cerevisiae* [18]. These studies led us to investigate the impact of overexpression of the two types of ALS for diacetyl production. In the present study, the ALS from *B. subtilis* was demonstrated to be more suitable for increased diacetyl production than the native ALS (Figure 2). One reason for this is the stronger preference of the ALS from *B. subtilis* for pyruvate over 2-ketobutyrate, when compared with that of the native ALS [19]. In addition, the native ALS (Ilv2p) is synthesized in the cytosol as a precursor protein with an N-terminal mitochondrial targeting sequence, which directs import into the mitochondrial matrix [20]. However, the expressed ALS from *B. subtilis* remains in the cytosol, and this cytosolic localization of ALS could lead to elevated diacetyl production [21].

In yeasts, the precursor of diacetyl, α-acetolactate, can be channeled to L-valine and L-leucine biosynthesis through acetohydroxy acid isomeroreductase encoded by the gene *ILV5*. Previous studies have reported that overexpression or cytosolic localization of the *ILV5* gene could limit diacetyl formation [22], [23]. Accordingly, in the present study, the gene *ILV5* was deleted and its suitability for increased diacetyl production was evaluated. It should be noted that Ilv5p is a bifunctional mitochondrial enzyme required for branched-chain amino acid biosynthesis as well as for the stability of mitochondrial DNA (mtDNA) and its parsing into nucleoids [24], [25]. It has been reported that the wild-type mtDNA is unstable in Δ*ilv5* mutant, leading to the production of ρ^−^ petites [26]. In the present study, strain DA-2 (Δ*ilv5*) showed a distinct decrease in the cell growth rate and maximum dry cell weight (18.8%, Figure 3B and 3C), although sufficient amounts of branched-chain amino acid and calcium pantothenate were added to the culture. This finding indicated that the stability of mtDNA could be affected by *ILV5* mutation in strain DA-2. Furthermore, deletion of the *ILV5* gene was found to increase the accumulation of α-acetolactate by 37% and diacetyl production by 28% (Table 2). This result suggested that the L-valine and L-leucine biosynthesis was a notable pathway in strain DA-1, which competed with diacetyl for α-acetolactate. It has been reported that diacetyl is reabsorbed by the yeast cells and converted to acetoin and subsequently to 2,3-butanediol by the action of BDH and other not fully characterized ketoreductase(s) [27], [28]. Two genes, *BDH1* and *BDH2*, encoding BDH have been characterized in *S. cerevisiae*, but only one gene has been detected in *C. glabrata* through gene annotation [10], [29]. Thus, *C. glabrata* may probably have other independent genes encoding BDH. In the present study, we showed that the *BDH* gene product is a major enzyme for DR and BDH activities in *C. glabrata*, and its disruption could lead to obvious limitation of diacetyl catabolism (Table 2).

Further improvement in the diacetyl-producing strain was achieved by enhancing the conversion of α-acetolactate to diacetyl in the presence of Fe^3+^. Most of the engineered strains described in previous studies have been reported to exhibit co-production of α-acetolactate and diacetyl following an inefficient NOD of α-acetolactate to diacetyl [4], [6]. In the study by Hugenholtz et al. [6], the final molar ratio of α-acetolactate and diacetyl was 57∶16, and the authors suggested that a more efficient chemical conversion of α-acetolactate to diacetyl could be achieved by extended aeration of the culture medium. In the present study, batch cultures of strain DA-3 were carried out in a 3-L fermentor and the impact of different dissolved oxygen concentrations on diacetyl production was studied. However, the results did not meet the expectations because of the increased volatilization of diacetyl (Table S2). Mohra et al. [12] reported that Cu^2+^ caused more significant decarboxylation of α-acetolactate to diacetyl than Fe^2+^ and Fe^3+^ during steam distillation of milk at pH 3.5. A complex between enediol, formed after decarboxylation of α-acetolactate, and a Cu^2+^–O_2_ complex may be involved in the Cu- or Fe-catalyzed diacetyl formation [30]. However, in the fermentation process, not only the ability of metal ions to transform α-acetolactate to diacetyl, but also their influence on cell growth, pyruvate accumulation, and the critical enzymes for diacetyl production should be considered. In the present study, we showed that Fe^3+^ could be a suitable accelerator for NOD of α-acetolactate to diacetyl, without causing obvious inhibition to cell growth or ALS activity (Figure 5).

The production of diacetyl by *C. glabrata* was enhanced stepwise, but the carbon flux channeled into the diacetyl biosynthetic pathway accounted for a very low proportion of the total carbon flux (Table 3). In the multi-vitamin auxotrophic *C. glabrata*, it was observed that the vitamin level in the culture medium could affect the activities of the corresponding enzymes (Figure 1), which also provided a strategy for redirecting the carbon flux by altering the vitamin level [31]. As shown in Table S1, thiamine is a cofactor for ALS, PDHc, ketoglutarate dehydrogenase, and PDC, while NA is involved in the TCA cycle and ethanol formation, but not in diacetyl biosynthesis. In the present study, the thiamine level was proved to be a key factor in redirecting the carbon flux from pyruvate to diacetyl. Increasing the thiamine level also resulted in increased yields of biomass and ethanol (Table 3), suggesting that more carbon flux was channeled into the TCA cycle and ethanol pathway. At this stage, a reduction in the NA level was expected to downregulate the TCA cycle and ethanol pathway, and increase the flux through diacetyl biosynthetic pathway. As expected, strain DA-3 showed obvious decrease in the biomass yield, but further increase in the diacetyl yield because of a lower NA level (Table 3). In addition, we also found that the decreased NA level led to a continuous increase in ethanol accumulation with improved NADH/NAD^+^ ratio (Tables 3 and 4). Nevertheless, the diacetyl titer increased from 2.1 to 4.7 g L^−1^, suggesting that redistribution of carbon flux at the pyruvate node was successful achieved by altering the thiamine and NA levels for increased diacetyl production.

In summary, metabolic engineering based on diacetyl metabolic pathway allowed for the development of *C. glabrata* capable of producing diacetyl. Furthermore, re-optimization of the vitamin level was also found to be necessary for redistributing the carbon flux into the desired pathway and realizing metabolic engineering goals. In the present study, a diacetyl yield of 0.05 g (g glucose)^−1^ was obtained, and further investigations are required to develop the engineered strain for industrial-scale production of diacetyl. The remaining DR and BDH activities in strain DA-3, which does not possess *BDH*, resulted in the reduction of diacetyl to acetoin and 2,3-butanediol, and the reduction was more obvious in the presence of higher diacetyl production (Table 3). Therefore, for optimal production of diacetyl, a more efficient inactivation of this reduction should be achieved. This may be accomplished by overexpressing NOX, which could lead to prevention of NADH-dependent reactions, such as acetoin and 2,3-butanediol formation as well as ethanol formation [6], [32].

## Materials and Methods

### Strains, Plasmids, and Genetic Modifications

The strains and plasmids used in this study are listed in Table 5. The primers used for gene cloning and deletion are listed in Table S3. The *ILV2* and *alsS* genes were respectively overexpressed with a *C. glabrata*-specific expression plasmid pYES-PGK1, which was constructed in the frame of plasmid pYES2 by replacing the *GAL1* promoter with *C. glabrata PGK1* promoter (Figure S1). The *ILV5* gene deletion in *C. glabrata* strain DA-1 was performed by homologous recombination, according to the method previously described [33], which comprised the following steps: construction of the *ILV5* deletion frame, efficient electroporation, enrichment with nystatin, and selection on limited medium (Figure S2). Inactivation of the *BDH* gene was achieved by replacing the *BDH* ORF with *ARG8* (Figure S3). The detailed procedures for genetic modifications are presented in the File S1.

**Table 5:** *Candida glabrata* strains and plasmids used in this study.

Strains/plasmids	Genotypes/descriptions	Sources
Strains		
FMME019	*C. glabrata* Δ*ura3* Δ*arg8* mutant strain derived from *C. glabrata* CCTCC M202019	[33]
DA-0	FMME019 pYES-PGK1	This study
DA-ILV2	FMME019 pYES-PGK1-ILV2	This study
DA-1	FMME019 pYES-PGK1-alsS	This study
DA-2	FMME019 Δ*ilv5* pYES-PGK1-alsS	This study
DA-3	FMME019 Δ*ilv5* Δ*bdh::arg8* pYES-PGK1-alsS	This study
Plasmids		
pYES2	2μ, *Amp*, *URA3*, P*_GAL1_*	Invitrogen
pYES-PGK1	2μ, *Amp*, *URA3*, P*_PGK1_*	This study
pYES-PGK1-ILV2	*C. glabrata ILV2* gene under the control of *PGK1* promoter in pYES-PGK1	This study
pYES-PGK1-alsS	*Bacillus subtilis alsS* gene under the control of *PGK1* promoter in pYES-PGK1	This study

### Media Formulations

Yeast peptone dextrose culture medium (YPD), nitrogen-free minimal medium (NFMM), minimal medium (MM), limited medium (LM), and supplement medium (SM) were used for the construction of the *C. glabrata* engineered strains, and their compositions were as follows: YPD (g L^−1^): yeast extract, 10; peptone, 20; and dextrose, 20. NFMM (g L^−1^): glucose, 20; KH_2_PO_4_, 1.0; and MgSO_4_, 0.5. MM: NFMM with 10 g L^−1^ of (NH_4_)_2_SO_4_. LM: MM with 5 mg L^−1^ of branched-chain amino acids (L-valine, L-leucine, and L-isoleucine) and 3 mg L^−1^ of calcium pantothenate. SM: MM with 40 mg L^−1^ of arginine (SM-A; for Δ*arg8* mutant); MM with 80 mg L^−1^ of uracil and 40 mg L^−1^ of arginine (SM-UA; for Δ*ura3* Δ*arg8* mutant); MM with 80 mg L^−1^ of branched-chain amino acids (valine, leucine, and isoleucine) and 10 mg L^−1^ of calcium pantothenate (SM-BP; for Δ*ilv5* mutant); and MM with 40 mg L^−1^ of arginine, 80 mg L^−1^ of branched-chain amino acids, and 30 mg L^−1^ of calcium pantothenate (SM-ABP; for Δ*arg8* Δ*ilv5* mutant).

The medium used for seed culture (medium A) was the corresponding SM according to the genotypes of the engineered strains. For slant culture, 2% agar was added to medium A. The fermentation medium (medium B) contained 100 g L^−1^ of glucose, 7 g L^−1^ of urea, 5 g L^−1^ of KH_2_PO_4_, 0.8 g L^−1^ of MgSO_4_·7H_2_O, and 3 g L^−1^ of sodium acetate. In addition, arginine (0.3 g L^−1^), branched-chain amino acids (0.5 g L^−1^), and calcium pantothenate (20 mg L^−1^) were added to the medium to overcome auxotrophy when necessary. The metal ion solutions were prepared as concentrated solutions and diluted in the corresponding ratio before use. The initial pH of all the media was adjusted to 5.5, and 10 mL of vitamin solution (8 mg L^−1^ of NA, 0.02 mg L^−1^ of thiamine, 0.04 mg L^−1^ of biotin, and 0.4 mg L^−1^ of pyridoxine-HCl) were added to all the media. All the amino acids, vitamins, and metal ion solutions were filter-sterilized before being added to the medium. All plates contained the corresponding liquid medium with 15 g L^−1^ of agar.

### Culture Conditions

The seed culture inoculated from the slant was cultivated in a 250-mL flask containing 25 mL of medium A on a reciprocal shaker (200 rpm, 30°C) for 24 h. Fermentation was carried out in a 500-mL flask containing 50 mL of medium B. The inoculum's size was 10% (v/v). For flask culture, 40 g L^−1^ of CaCO_3_ was used as the pH buffer after dry-heat sterilization at 160°C for 30 min. All cultivations were carried out at 30°C under agitation at 200 rpm in a shaker (HYL-B, Taicang, China).

### Quantification of Substrates and Products

Dry cell weight (DCW) was determined according to the method described by Liu et al. [34]. Glucose, pyruvate, ethanol, acetoin, and 2,3-butanediol were analyzed by high-performance liquid chromatography (HPLC) using a Dionex Ultimate 3000 series instrument equipped with a refractive index detector (RID, Agilent 1100 series). Analyte separation was achieved by using an Aminex® HPX-87H column (Bio-Rad, Hercules, CA, USA) with 5 mM H_2_SO_4_ as the mobile phase at a flow rate of 0.6 mL min^−1^. The column and detector temperatures were each set to 35°C throughout the analysis.

Determination of α-acetolactate and diacetyl was accomplished as described in previous reports [12]. In order to restrict spontaneous decarboxylation of α-acetolactate to diacetyl, the pH of the samples was adjusted to 0.5 or below using 4 M H_2_SO_4_ before the analysis. The samples were then examined using headspace gas chromatography with flame ionization detector (HS-GC-FID). During headspace at 70°C for 30 min in a low-pH environment, α-acetolactate was converted to acetoin, while diacetyl remained unchanged. Thus, α-acetolactate was quantified from the difference in the concentration of acetoin between the GC and HPLC results. HS-GC-FID analyses were performed using a gas chromatograph (GC-2010; Shimadzu Co., Kyoto, Japan), and analyte separation was accomplished on a capillary column (PEG-20M, 30 m×0.32 mm I.D.). Nitrogen was used as the carrier gas at a flow rate of 1.2 mL min^−1^. The injection and detector temperatures were 200°C and 250°C, respectively, and the temperature program was as follows: 5 min at 40°C, subsequent increase to 180°C at the rate of 10°C min^−1^, and 5 min at 180°C.

The amino acids were separated by Agilent 1200 HPLC using a 4.0 125-mm Hypersil ODS C18 column. The solvents and gradient conditions employed were as described by Chen et al. [35]. The detection wavelength was set at UV 338 nm. The extraction and detection of intracellular NADH and NAD^+^ were carried out as described previously [36].

### Quantification of Enzyme Activity

The cell extracts for enzyme activity assay were prepared as previously described [34]. The activities of ALS from *C. glabrata* [37] and *B. subtilis* [38] were determined as described earlier. The DR and BDH activities were assayed with diacetyl and acetoin as substrates, respectively, as previously reported [39]. The protein concentration was measured by the Lowry method, with bovine serum albumin as the standard [40].

## Supporting Information

File S1 **The detailed procedures for genetic modifications.**
(DOCX)

Figure S1 **Construction and confirmation of the ALS expressed strains.** (A) The construction of expression expression plasmid pYES-PGK1; (B) Analysis of recombinant plasmid pYES-PGK1 with restriction endonulease digestion; (C) Confirmation of positive clones overexpressing *ILV2* by restriction endonulease digestion; (D) Colony PCR of positive clones overexpressing alsS. Lane M, 10 kb Marker; Lane 1, pYES-PGK1 with *Hin*dIII and *Nae*I; Lane 2, pYES-PGK1-ILV2 with *Hin*dIII and *Xho*I; Lane 3, pYES-PGK1-ILV2; Lane 4, positive strains overexpressing *alsS*; Lane 5, control stain DA-0.
(DOCX)

Figure S2 **The knockout and confirmation of the** ***ILV5*** **gene.** (A) Construction of fusion frames used for gene deletion; (B) The schematics of gene knockouts; (C) Purified PCR fragments used for *ILV5* deletion; (D) Enrichment result of *ILV5* mutants on LM; (E) Confirmation of the auxotrophic mutants on SM-A and SM-ABP plates; (F) Colony PCR of the DA-2 positive clones. Lane M, 10 kb DNA Marker; Lane 1, *ILV5* knockout frame; Lane 2, *ILV5* left arm; Lane 3, *ILV5* right arm; Lane 4–5, strain DA-2; Lane 6, control stain DA-1.
(DOCX)

Figure S3 **The knockout and confirmation of the** ***BDH*** **gene.** (A)The construction of *BDH* knockout frame for *BDH* inactivation; (B) Purified PCR fragments used for *BDH* inactivation. Lane M, 10 kb DNA Marker; Lane 1, Δ*bdh::arg8* cassette; Lane 2, *BDH* left arm; Lane 3, *ARG8* ORF; Lane 4, *BDH* right arm; (C) The schematic of gene *BDH* knockout; (D) Colony PCR of the DA-3 positive clones. Lane 5–6, strain DA-3; Lane 7, control strain DA-2.
(DOCX)

Table S1 **The reactions with thiamine or NA as cofactor in the central metabolic pathway of** ***C. glabrata*** **.**
(DOCX)

Table S2 **Comparison of diacetyl production of strain DA-3 under different dissolved oxygen concentration.**
(DOCX)

Table S3 **Primers used in this study.**
(DOCX)
