# Supplementary material for: Metabolic Engineering of Candida glabrata for Diacetyl Production
Source: PLoS One. 2014 Mar 10;9(3):e89854. doi: 10.1371/journal.pone.0089854 (PMC3948628; doi:10.1371/journal.pone.0089854)
Supplement: File S1 — The detailed procedures for genetic modifications. (DOCX) [file pone.0089854.s001.docx]

Metabolic engineering of *Candida glabrata* for diacetyl production

*Fur publication in PLoS ONE*

Xiang Gao, Nan Xu, Shubo Li, Liming Liu

The detailed procedures for genetic modifications

Plasmids construction

1. For the construction of a *Candida glabrata*-specific expression plasmid pYES-PGK1, the *GAL1* promoter in the plasmid pYES2 was replaced with *C. glabrata PGK1* promoter. As shown in Figure S1A, the plasmid pYES2 was amplified by PCR using the primer combination f1ori-f/f1 ori-s (PCR1) and previously isolated genomic DNA of *C. glabrata* CCTCC M202019 was amplified by PCR using the combination PGK1-f/PGK1-r (PCR2). In a second step, the two PCR products were fused using the primer combination f1ori-f/ PGK1-r (PCR3). Subsequent to the amplification steps, the PCR-product was digested using restriction enzymes *Nae*I and *Hind*III and inserted into the plasmid pYES2 using T4 DNA ligase. The resulting pYES-PGK1 was then validated by restriction enzyme digestion (Figure S1B) and sequencing.
2. For amplification of the *ILV2* gene, encoding acetolactate synthase, previously isolated genomic DNA of *C. glabrata* was amplified by PCR using the primer combination ilv2-f/ilv2-r. The PCR-product was cut using restriction enzymes *Hind*III and *Xho*I and inserted into the plasmid pYES-PGK1 which was subsequently used to transform *E. coli* JM109. The resulting plasmid pYES-PGK1-ILV2 was isolated from JM109 and validated by sequencing.
3. For amplification of the *alsS* gene, encoding acetolactate synthase from *Bacillus subtilis*, the isolated genomic DNA of *B. subtilis* was amplified by PCR using the primer combination alsS-f/alsS-r. The PCR-product was cut using restriction enzymes *BamH*I and *Sph*I and cloned into the plasmid pYES-PGK1 which was subsequently used to transform *E. coli* JM109. The resulting plasmid pYES-PGK1-alsS was also validated by sequencing.

Electroporation and confirmation

1. The constructed plasmids were transformed into *C. glabrata* cells via electroporation as follows. Cultures were grown to saturation overnight in YPD. The next morning, a 100 mL culture of YPD was inoculated to OD600 = 0.1. Inoculated cells were grown on a reciprocal shaker at 200 rpm and 30 ^o^C until OD600 = 1.5 after 8h. Cells were collected via centrifugation at 3000 rpm for 3 min and the media removed. The cell pellet was washed once by 20 mL ice-cold water. The cells were re-suspended with 20 mL ice-cold solution containing 10 mM Tris-HCl, 1mM EDTA, 25 mM Dithiothreitol (DTT), and 400 mM lithium acetate and placed on reciprocal shaker for 1 h at 50 rpm and 30 ^o^C. Conditioned cells were collected by centrifugation and washed twice by 20 mL ice-cold water, and then washed once by 10 mL ice-cold 1M sorbitol. The cell pellet was re-suspended to a final volume of 1 mL in ice-cold 1M sorbitol. In all, 80 μL of cells were used per electroporation with 5 μg plasmid DNA (the pYES-PGK1-ILV2 and pYES-PGK1-alsS, respectively). Cells were electroporated at 2.5 kV, 25 μF, 200 Ω using a GenePulser Xcell^TM^ electroporation system (BioRad, Herculus, USA). Electroporated cells were immediately diluted with 1 mL of ice-cold 1M sorbitol and cultured at 30 ^o^C for 2h. Approximately 400 μL cells were spread on SM-A plates to identify Ura^+^ prototrophic colonies.
2. For confirmation of the colonies which has plasmid pYES-PGK1-ILV2, the colonies were inoculated into 25 mL of YPD and grown on a reciprocal shaker (200 rpm, 30 ^o^C) for 24 h. Approximately 2 mL cells were collected via centrifugation, and then plasmid isolation was performed with kit from Qiagen (Hilden, Germany). Only trace amounts of plasmid could be isolated from yeast, therefore the plasmid DNA amplification was necessary. To this end, the isolated plasmid from yeast was transformed into *E. coli* JM109. The plasmid was isolated from JM109 and validated by restriction enzyme digestion (Figure S1C) and sequencing. The positively identified mutant was used for subsequent experiments.
3. For confirmation of the colonies which has plasmid pYES-PGK1-alsS, Colony PCR was performed as follows. A small amount of cells was scraped from a colony, transferred to the wall of a 1.5 mL-Eppendorf tube and heated in a microwave oven for 1 min (600 W). The treated cells were subsequently amplified using PCR with primer combination alsS-f/alsS-r (Figure S1D). A positively identified mutant, in the following referred to as *C. glabrata* DA-1, was used for subsequent genetic modifications.

Gene deletion

1. For deletion of the *ILV5* gene, encoding acetohydroxy acid reductoisomerase, previously isolated genomic DNA of *C. glabrata* was amplified with the primer combinations ilv5-left-f/ilv5-left-r and ilv5-right-f/ilv5-right-r, respectively. In a second step, the two PCR products were fused using the primer combination ilv5-left-f/ ilv5-right-r (Figure S2A and S2C). The PCR-product was validated by sequencing and then transformed into *C. glabrata* DA-1 competent cells. The transformed cells were grown in 25 mL (250 mL flask) of YPD for 24 h (200 rpm, 30 ^o^C) and then washed (3000 rpm for 3 min) twice by NFMM and grown in 25 mL NFMM as above, to exhaust intracellular nitrogen source (Approximately 6 h). The resulting cells were washed with SM-A twice, as above, and grown in 25 mL SM-A until the OD600 doubled. The culture was supplemented with nystatin (10 μg/mL) and sucrose (170 g/L), then static cultured at 30 ^o^C in a thermostat-controlled water-bath with circulating water for 90 min. The flask was gently rotated every 15 min to prevent sedimentation of cells. Nystatin can bind to ergosterol, a major component of the fungal cell membrane. When cells begin to grow, the binding sites on membrane would be disrupted and lead to cell death. The *ILV5* mutant was auxotrophic for branched amino acid and pantothenate and cannot grow in SM-A, therefore, it can survive after this process, which is called nystatin enrichment. In order to remove the nystatin, cells were washed twice with MM. The cells were then diluted with MM and spread on LM plates containing trace amount of branched amino acid and calcium pantothenate (Figure S2D). The smaller colonies that appeared on LM after 72 h were transformed to corresponding SM-A and SM-ABP plates. The colonies that grow on SM-ABP but not on SM-A were confirmed by streaking at least three times on SM-A and SM-ABP, and by colony PCR (Figure S2E and S2F). A positively identified mutant, in the following referred to as *C. glabrata* DA-2, was used for subsequent genetic modifications.
2. For deletion of the *BDH* gene, encoding diacetyl reductase, previously isolated genomic DNA of *C. glabrata* was amplified with the primer combinations bdh-left-f/bdh-left-r (PCR1), arg8ORF-f/arg8ORF-r (PCR2), and bdh-right-f/bdh-right-r (PCR3) (Figure S3A and S3B). In PCR1 and PCR3, upstream sequence and downstream sequence of the *BDH* gene were amplified respectively. In PCR2 the *ARG8* ORF was amplified, and overlapping sequences with the upstream sequence and downstream sequence of the *BDH* gene were artificially added at the 5’-end and 3’-end, respectively. In the next step, these three DNA fragments were fused in PCR4 using primers bdh-left-f and bdh-right-r. The resulting fragment was validated by sequencing and then transformed into *C. glabrata* DA-2 competent cells. The transformed cells were spread on SM-A plates to identify Arg^+^ prototrophic colonies, and the colonies were confirmed by colony PCR (Figure S3D). A positively identified mutant will in the following be referred to as *C. glabrata* DA-3.
