# Supplementary material for: Metabolic Engineering of Candida glabrata for Diacetyl Production
Source: PLoS One. 2014 Mar 10;9(3):e89854. doi: 10.1371/journal.pone.0089854 (PMC3948628; doi:10.1371/journal.pone.0089854)
Supplement: Figure S1 — Construction and confirmation of the ALS expressed strains. (A) The construction of expression expression plasmid pYES-PGK1; (B) Analysis of recombinant plasmid pYES-PGK1 with restriction endonulease digestion; (C) Confirmation of positive clones overexpressing ILV2 by restriction endonulease digestion; (D) Colony PCR of positive clones overexpressing alsS. Lane M, 10 kb Marker; Lane 1, pYES-PGK1 with HindIII and NaeI; Lane 2, pYES-PGK1-ILV2 with HindIII and XhoI; Lane 3, pYES-PGK1-ILV2; Lane 4, positive strains overexpressing alsS; Lane 5, control stain DA-0. (DOCX) [file pone.0089854.s002.docx]

Supplementary Figures


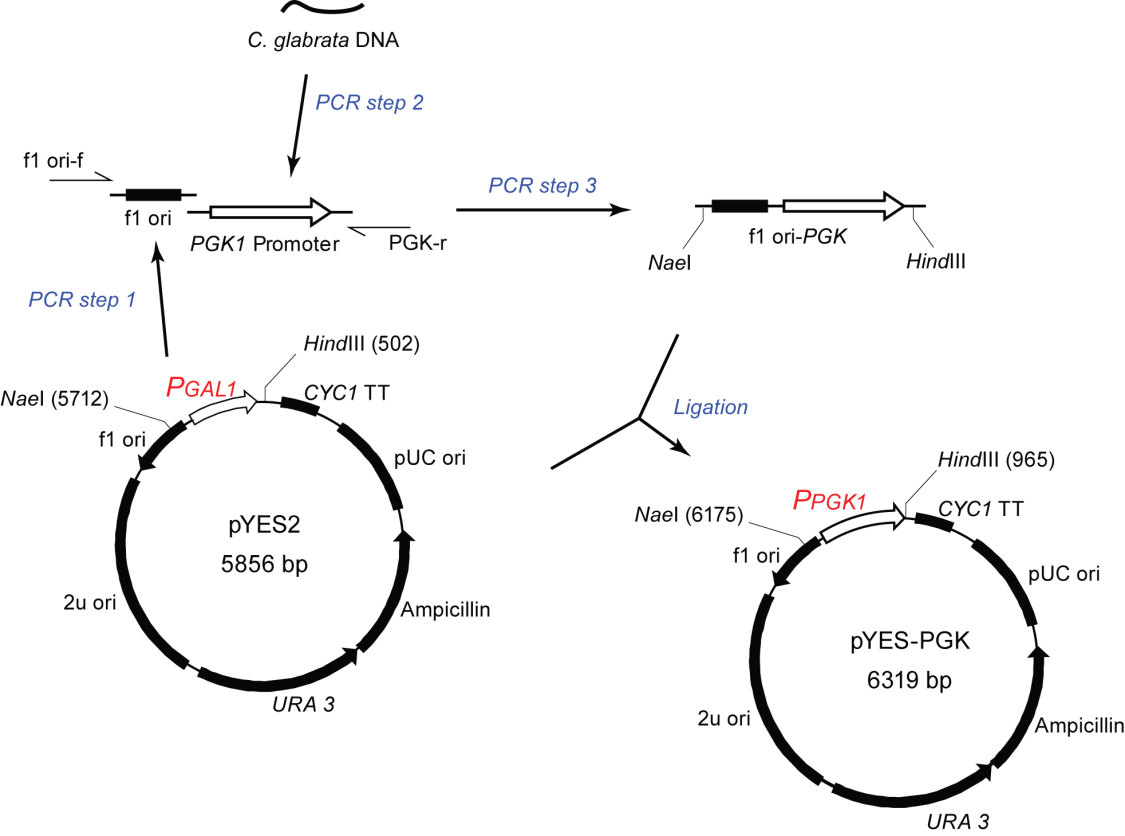


(B)

(A)

(D)

(C)


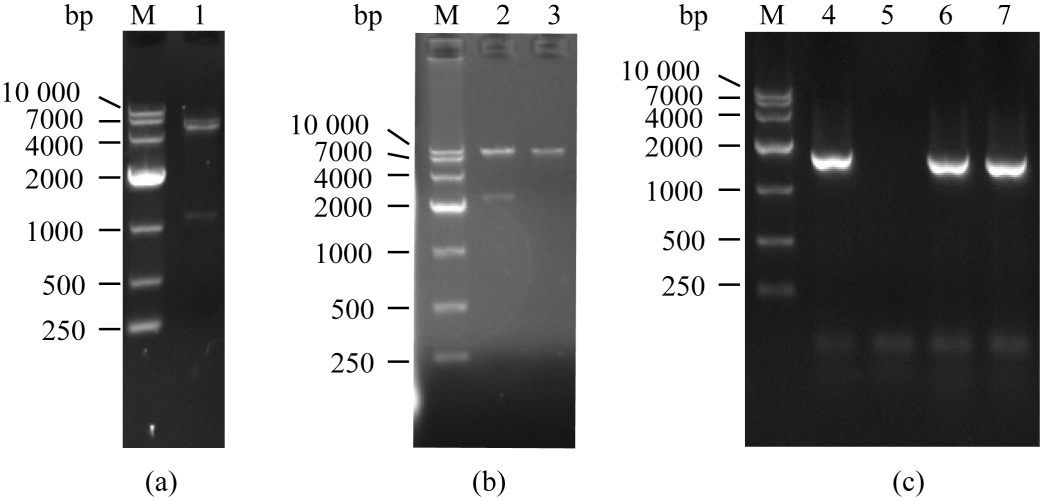


Figure S1 Construction and confirmation of the ALS expressed strains. (A) The construction of expression expression plasmid pYES-PGK1; (B) Analysis of recombinant plasmid pYES-PGK1 with restriction endonulease digestion; (C) Confirmation of positive clones overexpressing *ILV2* by restriction endonulease digestion; (D) Colony PCR of positive clones overexpressing *alsS*. Lane M, 10 kb Marker; Lane 1, pYES-PGK1 with *Hind*III and *Nae*I; Lane 2, pYES-PGK1-ILV2 with *Hind*III and *Xho*I; Lane 3, pYES-PGK1-ILV2; Lane 4, positive strains overexpressing *alsS*; Lane 5, control stain DA-0.
