# Supplementary material for: Metabolic Engineering of Candida glabrata for Diacetyl Production
Source: PLoS One. 2014 Mar 10;9(3):e89854. doi: 10.1371/journal.pone.0089854 (PMC3948628; doi:10.1371/journal.pone.0089854)
Supplement: Figure S2 — The knockout and confirmation of the ILV5 gene. (A) Construction of fusion frames used for gene deletion; (B) The schematics of gene knockouts; (C) Purified PCR fragments used for ILV5 deletion; (D) Enrichment result of ILV5 mutants on LM; (E) Confirmation of the auxotrophic mutants on SM-A and SM-ABP plates; (F) Colony PCR of the DA-2 positive clones. Lane M, 10 kb DNA Marker; Lane 1, ILV5 knockout frame; Lane 2, ILV5 left arm; Lane 3, ILV5 right arm; Lane 4–5, strain DA-2; Lane 6, control stain DA-1. (DOCX) [file pone.0089854.s003.docx]

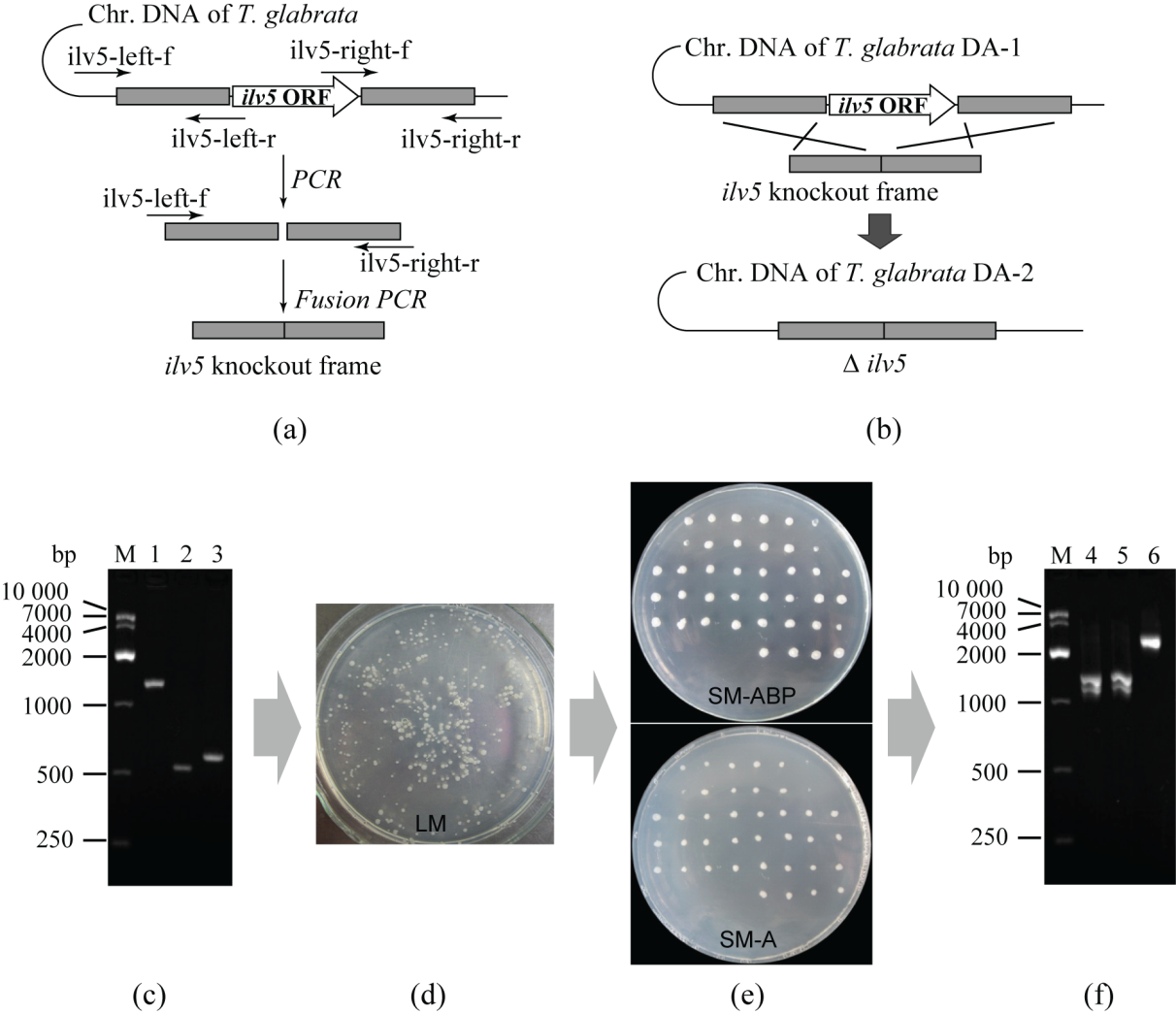


(E)

(F)

(D)

(C)

(B)

(A)

Figure S2 The knockout and confirmation of the *ILV5* gene. (A) Construction of fusion frames used for gene deletion; (B) The schematics of gene knockouts; (C) Purified PCR fragments used for *ILV5* deletion; (D) Enrichment result of *ILV5* mutants on LM; (E) Confirmation of the auxotrophic mutants on SM-A and SM-ABP plates; (F) Colony PCR of the DA-2 positive clones. Lane M, 10 kb DNA Marker; Lane 1, *ILV5* knockout frame; Lane 2, *ILV5* left arm; Lane 3, *ILV5* right arm; Lane 4-5, strain DA-2; Lane 6, control stain DA-1.
