# Supplementary material for: Metabolic Engineering of Candida glabrata for Diacetyl Production
Source: PLoS One. 2014 Mar 10;9(3):e89854. doi: 10.1371/journal.pone.0089854 (PMC3948628; doi:10.1371/journal.pone.0089854)
Supplement: Figure S3 — The knockout and confirmation of the BDH gene. (A)The construction of BDH knockout frame for BDH inactivation; (B) Purified PCR fragments used for BDH inactivation. Lane M, 10 kb DNA Marker; Lane 1, Δbdh::arg8 cassette; Lane 2, BDH left arm; Lane 3, ARG8 ORF; Lane 4, BDH right arm; (C) The schematic of gene BDH knockout; (D) Colony PCR of the DA-3 positive clones. Lane 5–6, strain DA-3; Lane 7, control strain DA-2. (DOCX) [file pone.0089854.s004.docx]

control stain DA-1.


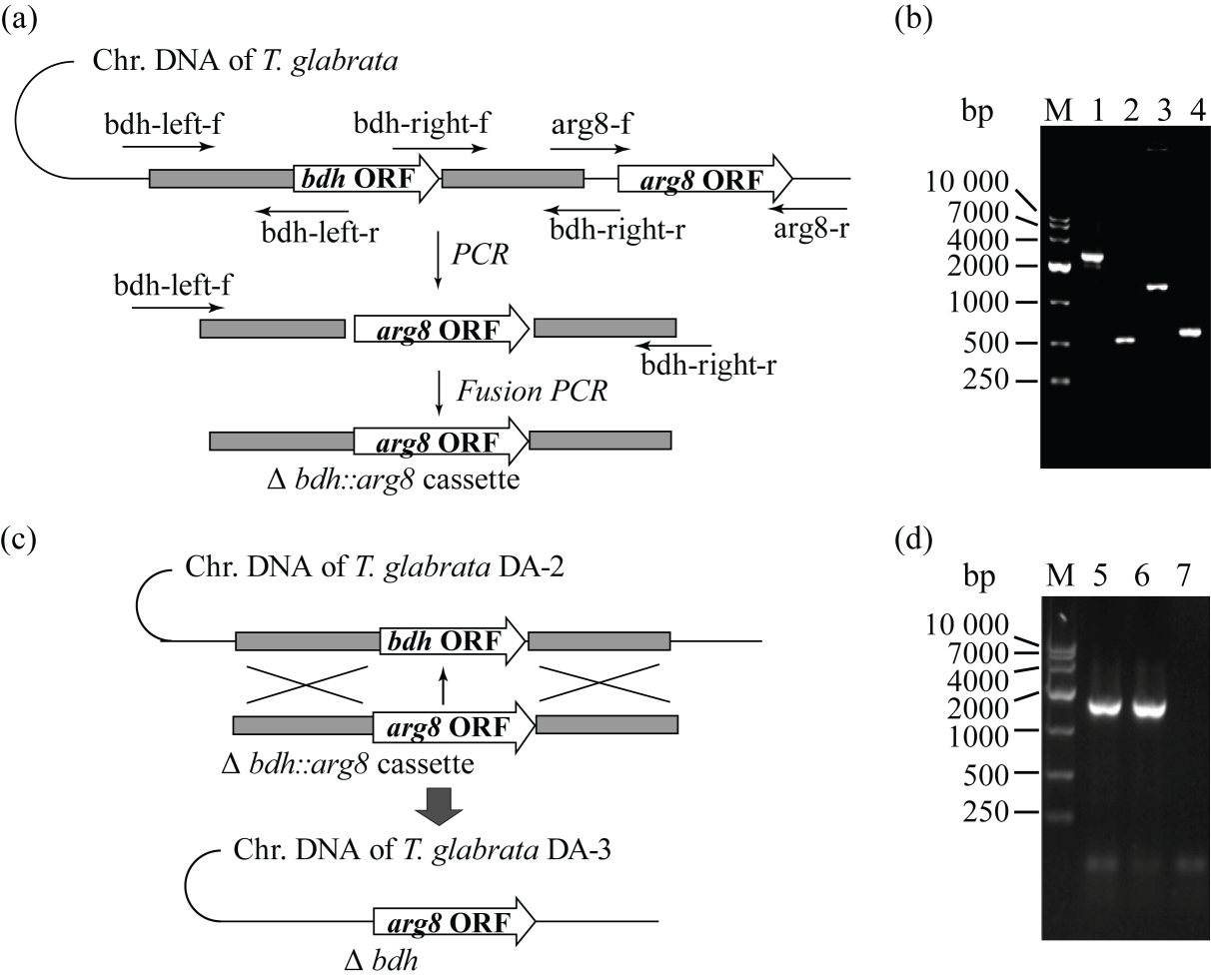


(D)

(C)

(B)

(A)

Figure S3 The knockout and confirmation of the *BDH* gene. (A)The construction of *BDH* knockout frame for *BDH* inactivation; (B) Purified PCR fragments used for *BDH* inactivation. Lane M, 10 kb DNA Marker; Lane 1, Δ*bdh::arg8* cassette; Lane 2, *BDH* left arm; Lane 3, *ARG8* ORF; Lane 4, *BDH* right arm; (C) The schematic of gene *BDH* knockout; (D) Colony PCR of the DA-3 positive clones. Lane 5-6, strain DA-3; Lane 7, control strain DA-2.
