# Supplementary material for: Metabolic Engineering of Candida glabrata for Diacetyl Production
Source: PLoS One. 2014 Mar 10;9(3):e89854. doi: 10.1371/journal.pone.0089854 (PMC3948628; doi:10.1371/journal.pone.0089854)
Supplement: Table S1 — The reactions with thiamine or NA as cofactor in the central metabolic pathway of C. glabrata . (DOCX) [file pone.0089854.s005.docx]

Supplementary Tables

Table S1 The reactions with thiamine or NA as cofactor in the central metabolic pathway of *C. glabrata*.

| **Subsystem** | **Reaction equation** | **Enzyme** | **Thiamine** | **NA** |
| --- | --- | --- | --- | --- |
| Diacetyl biosynthesis | PYR[m] + TPP[m] <=> 2HTPP[m] + CO2[m] | Acetolactate synthase | **√** |  |
|  | 2HTPP[m] + PYR[m] + H[m] <=> ACLAC[m] + TPP[m] | Acetolactate synthase | **√** |  |
|  | ACLAC[m] <=> ACLAC[c] | - |  |  |
|  | ACLAC[c] <=> ACLAC[e] | - |  |  |
|  | ACLAC[e] + O2[e] + 3H[e] <=> DACE[e] + CO2[e] + 2H2O[e] | Non-enzymatic decarboxylation |  |  |
|  |  |  |  |  |
| Glycolysis | T3P1[c] + PI[c] + NAD[c] <=> 13PDG[c] + NADH[c] + H[c] | Glycerol-3-phosphate dehydrogenase |  | **√** |
|  |  |  |  |  |
| TCA cycle | PYR[m] + TPP[m] <=> 2HTPP[m] + CO2[m] | Pyruvate dehydrogenase complex | **√** |  |
|  | 2HTPP[m] + LIPO[m] + H[m] <=> DHACAHP[m] + TPP[m] | Pyruvate dehydrogenase complex | **√** |  |
|  | DHACAHP[m] + COA[m] + NAD[m] <=> ACCOA[m] + LIPO[m] + NADH[m] | Pyruvate dehydrogenase complex |  | **√** |
|  | ICIT[m] + NAD[m] <=> CO2[m] + NADH[m] + AKG[m] | Isocitrate dehydrogenase |  | **√** |
|  | MAL[m] + NAD[m] <=> OAA[m] + NADH[m] + H[m] | Malate dehydrogenase |  | **√** |
|  | AKG[m] + TPP[m] + H[m] <=> 3C1HTHPP[m] + CO2[m] | Ketoglutarate dehydrogenase | **√** |  |
|  | 3C1HTHPP[m] + LIPO[m] <=> DISUCLY[m] + TPP[m] | Ketoglutarate dehydrogenase | **√** |  |
|  |  |  |  |  |
| Ethanol biosynthesis | PYR[m] + TPP[m] <=> 2HTPP[m] + CO2[m] | Pyruvate decarboxylase | **√** |  |
|  | 2HTPP[m] + H[m] <=> ACAL[m] + TPP[m] | Pyruvate decarboxylase | **√** |  |
|  | ACAL[c] + NADH[c] + H[c] <=> ETOH[c] + NAD[c] | Alcohol dehydrogenase |  | **√** |
