# Supplementary material for: Metabolic Engineering of Candida glabrata for Diacetyl Production
Source: PLoS One. 2014 Mar 10;9(3):e89854. doi: 10.1371/journal.pone.0089854 (PMC3948628; doi:10.1371/journal.pone.0089854)
Supplement: Table S2 — Comparison of diacetyl production of strain DA-3 under different dissolved oxygen concentration. (DOCX) [file pone.0089854.s006.docx]

Table S2 Comparison of diacetyl production of strain DA-3 under different dissolved oxygen concentration.

| Parameters | Flask | Fermentor (dissolved oxygen concentration) | | |
| --- | --- | --- | --- | --- |
|  |  | 20% | 50% | 80%*^a^* |
| Specific growth rate (h^-1^) | 0.12 | 0.11 | 0.13 | 0.15 |
| Biomass (g L^-1^) | 9.4 | 9.8 | 10.4 | 11.1 |
| α-Acetolactate (g L^-1^) | 0.86 | 0.68 | 0.64 | 0.52 |
| Diacetyl (g L^-1^) | 0.95 | 0.67 | 0.60 | 0.47 |
| Pyruvate (g L^-1^) | 36.7 | 38.9 | 43.5 | 53.6 |

*^a^* The aeration rate was increased to 2.5 vvm.

For evaluating the impact of different dissolved oxygen (DO) concentrations on diacetyl production, batch cultures of strain DA-3 were performed in a 3 L fermentor (BioFlo/CelliGen 115-3 L; New Brunswick Scientific, Enfield, CT, USA), with 2 L of fermentation medium. The inoculation size was 10% (v/v). The pH of the culture medium was adjusted to 5.5 with 8 mol L^-1^ NaOH. The cultures were maintained at 30 ^o^C with an aeration rate of 1.5 vvm. The DOT was measured with DO probe and controlled at a constant value by a proportional-integral-derivative (PID) algorithm that varied the agitation rate.

As shown in Table S2, with increasing DO concentration in the culture, strain DA-3 showed increased growth rate and pyruvate production, but showed decrease in both the α-acetolactate and diacetyl production. We thought one reason was the increased volatilization of diacetyl. And this hypothesis was subsequently proved by the detection of diacetyl in the solution that absorbed the exhaust gas from fermentor. Under a DO concentration of 80%, at least 0.35 g L^-1^ of diacetyl (computed data according the volume of culture medium and the solution) was detected in the solution that absorbed exhaust gas. Our results indicated that extended aeration of the culture medium may be a disadvantage for diacetyl accumulation during fermentation.
