# Supplementary material for: Metabolic Engineering of Candida glabrata for Diacetyl Production
Source: PLoS One. 2014 Mar 10;9(3):e89854. doi: 10.1371/journal.pone.0089854 (PMC3948628; doi:10.1371/journal.pone.0089854)
Supplement: Table S3 — Primers used in this study. (DOCX) [file pone.0089854.s007.docx]

Table S3 Primers used in this study.

| Primer name | Sequence (5’→3’) | Purpose |
| --- | --- | --- |
| f1 ori-f | GAAA**GCCGGC**GAACGTGGCGAGAAAGGAAG (*Nae*I) | f1 ori PCR |
| f1 ori-r | GGTGCAGGTGACTAGTGGATCATCCCCACGC | f1 ori PCR |
| PGK1-f | GGGATGATCCACTAGTCACCTGCACCAGCAACAAC | PGK1 PCR |
| PGK1-r | CCC**AAGCTT**TATCGAATAGATGTATGTATGCCGTCTTGC (*Hind*III) | PGK1 PCR |
|  |  |  |
| ilv2f | CCC**AAGCTT**ATGACGAGAAGAGGGCTAACGGAT (*Hind*III) | *ILV2* PCR |
| ilv2r | CCG**CTCGAG**CTAAAATTTGTTTGTCTTAATGCTT (*Xho*I) | *ILV2* PCR |
| alsSf | CG**GGATCC**ATGGACAAAAGCAACAAAAGAACAAA(*Bam*HI) | *alsS* PCR |
| alsSr | ACAT**GCATGC**CTAGAGAGCTTTCGTTTTCATGAG (*Sph*I) | *alsS* PCR |
|  |  |  |
| ilv5-left-f | GGACCACCTCTGTACTGATGTTATGTCATACCG | *ILV5* deletion |
| ilv5-left-r | AATTCAGATTCTAGCAGTTGTCCTGGTAGTGTTTGAAATC | *ILV5* deletion |
| ilv5-right-f | CTACCAGGACAACTGCTAGAATCTGAATTGCAAACC | *ILV5* deletion |
| ilv5-right-r | GCTCTACAGTTGCACTTTGCGTAATGCAGTTAG | *ILV5* deletion |
|  |  |  |
| bdh-left-f | AGCAGAGATAGCGGCGTTATGTAGTCGTGT | *BDH* deletion |
| bdh-left-r | TCTCTTGTACATCTTGTAATAGTTTGGTAAGCTGGAAC | *BDH* deletion |
| arg8ORF-f | CCAAACTATTACAAGATGTACAAGAGATATTTCTCCAC | *BDH* deletion |
| arg8ORF-r | CGTCTAGAGAGTACTTATTTTGAGAAGACGTCATTAACT | *BDH* deletion |
| bdh-right-f | CGTCTTCTCAAAATAAGTACTCTCTAGACGAAGTAATGATA | *BDH* deletion |
| bdh-right-r | AAGTTGACGATACCTATTGCGATGCGATG | *BDH* deletion |

The restriction sites in the primer sequences are shown in boldface. Overlapping sequences required for the fusion PCR are underlined.
